# Fracmemristor chaotic oscillator with multistable and antimonotonicity properties

**DOI:** 10.1016/j.jare.2020.05.025

**Published:** 2020-06-17

**Authors:** Haikong Lu, Jiri Petrzela, Tomas Gotthans, Karthikeyan Rajagopal, Sajad Jafari, Iqtadar Hussain

**Affiliations:** a School of Electronic Engineering, Changzhou College of Information Technology, 213164, China; b Department of Radio Electronics, Brno University of Technology, 616 00 Brno, Czech Republic; c Nonlinear Systems and Applications, Faculty of Electrical and Electronics Engineering, Ton Duc Thang University, Ho Chi Minh City, Viet Nam; d Department of Biomedical Engineering, Amirkabir University of Technology, 424 Hafez Ave., Tehran 15875-4413, Iran; e Department of Mathematics, Statistics and Physics, Qatar University, Doha 2713, Qatar

**Keywords:** Memristor, Fracmemristor, Chaotic oscillators, Multistability, Antimonotonicity

## Abstract

Memristor is a non-linear circuit element in which voltage-current relationship is determined by the previous values of the voltage and current, generally the history of the circuit. The nonlinearity in this component can be considered as a fractional-order form, which yields a fractional memristor (fracmemristor). In this paper, a fractional-order memristor in a chaotic oscillator is applied, while the other electronic elements are of integer order. The fractional-order range is determined in a way that the circuit has chaotic solutions. Also, the statistical and dynamical features of this circuit are analyzed. Tools like Lyapunov exponents and bifurcation diagram show the existence of multistability and antimonotonicity, two less common properties in chaotic circuits.

## Graphical abstract

**Figure:**
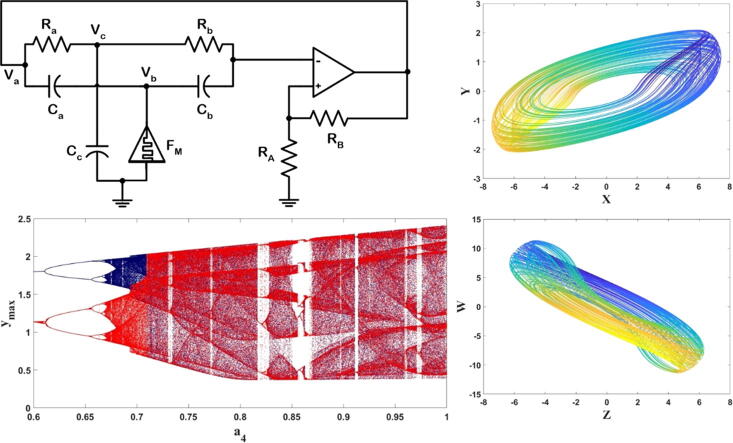

## Introduction

A memristor is a non-linear circuit circuit element, which is based on nonlinear voltage-current relation. The electrical resistance of this element is related to its previous current, so it has been named memristor (memory resistor) [1]. Circuits and systems containing memristors have been successfully used in image and text encryption, simulating biological systems, electronic and neural networks [2]. Continuous symmetrical, continuous nonsymmetrical, switching and fractional models of memristor with its emulators and realizations are discussed in [3]. Chaotic circuits and systems are interesting topics in nonlinear dynamics [4]. Various chaotic systems have been proposed in recent years [5], [6]. Memristive systems show complex dynamical behaviors, like chaos [7], multistability [8], and hidden attractors. Designing and analyzing memristive systems and circuits with particular properties have been considered in different oscillator e.g., Wien-bridge oscillator [9], diode bridge-based oscillator [10] and neuron models [11].

Fractional-order differential equations are in the group of nonlinear and complex systems [12], [13], [14]. These systems have shown different complex properties such as hyperchaos [15], self-producing attractors, and strange maps [16], which enabled them to be used in modeling of biological phenomena, electrical components, controllers, and filters [17]. Multistability and antimonotonicity are two features that have been reported in fractional-order systems [18]. The predictor–corrector method of the Adams-Bashforth-Moulton (ABM) algorithm can be used to discretize fractional-order equations, especially when systems are highly sensitive.

Several studies have been done recently to develop and realize the fractional-order element. Fractional parameters of these elements provide flexibility and degrees of freedom in computational modeling [19], control engineering [20], [21], and filter designing [22]. Although the fractional-order form of the three conventional elements has been explored well, studying this form of memristor still is a new topic. Step, DC, sinusoidal, and non-sinusoidal periodic responses of the fractional-order memristor have been analyzed in [23], [24]. Some researches show that saturation time of this element changes when fractional order and voltage change [23], [24]. Also, considering fractional order makes a charge-controlled memristor have two hysteresis loop in its V-I plane [25].To compare the effect of using fractional memristor, reference [26] shows that a wider range of frequency is generated using the memristor with fractional-order elements, rather than integer ones. Also, considering fractional-order memristive Chua’s circuit makes it a non-smooth system which shows different bifurcations such as tangent or grazing ones [27].

As fractional-systems are in the group of complex systems, they need relevant analyzing tools. To analyze the statistical properties of the systems, equilibria, eigenvalues, and stability should be checked. In these systems, the stability depends on the value of the order in addition to the eigenvalues. Also, to analyze the dynamical properties of the systems, Lyapunov exponents (LEs) shows the divergence of the adjacent initial conditions. Wolf’s algorithm [28] is a well-known algorithm that numerically estimates the LEs of the system. In that case, the positivity of the largest Lyapunov exponent (LLE) of the system shows the chaoticity of the system. The bifurcation diagram of the systems is another tool to analyze the attractors of the systems as the controlling parameter(s) changes. Using bifurcation diagram, one can explore the multistability and antimonotonicity of the system.

We completely introduce the fracmemristor and Twin-T oscillator mathematical model and circuit in Section 2. The statistical and dynamical properties of the proposed fractional-order model are analyzed in Section 3. We also explain the stability of the equilibriums, the Lyapunov exponents, bifurcation diagram, multistability, and antimonotonicity of the proposed model in that section. Finally, the conclusion of this work is presented in Section 4.

## Fracmemristor Twin-T oscillator (FTT)

The fractional-order form of the memristor is given by [24],

(1) $$R_{m}={\left[R_{\mathrm{in}}^{q+1}\mp \frac{\Gamma \left(q+2\right)}{\Gamma \left(q\right)}g\left(R_{\mathrm{on}}-R_{\mathrm{off}}\right)\underset{0}{\int^{t}}{\left(t-\tau \right)}^{q+1}\upsilon \left(\tau \right)d\tau \right]}^{\frac{1}{q+1}}$$

in which *R_m_*, *R_on_*, *R_off_* and Rin denote the moment, minimum, maximum, and initial value resistances of the memristor, respectively. Also, g and q are the memristor constant and the fractional-order which varies in the range of$\left(0,1\right)$. It should be noted that the memristor in (1) becomes integer-order, when q=1.

**Fig. 1:**
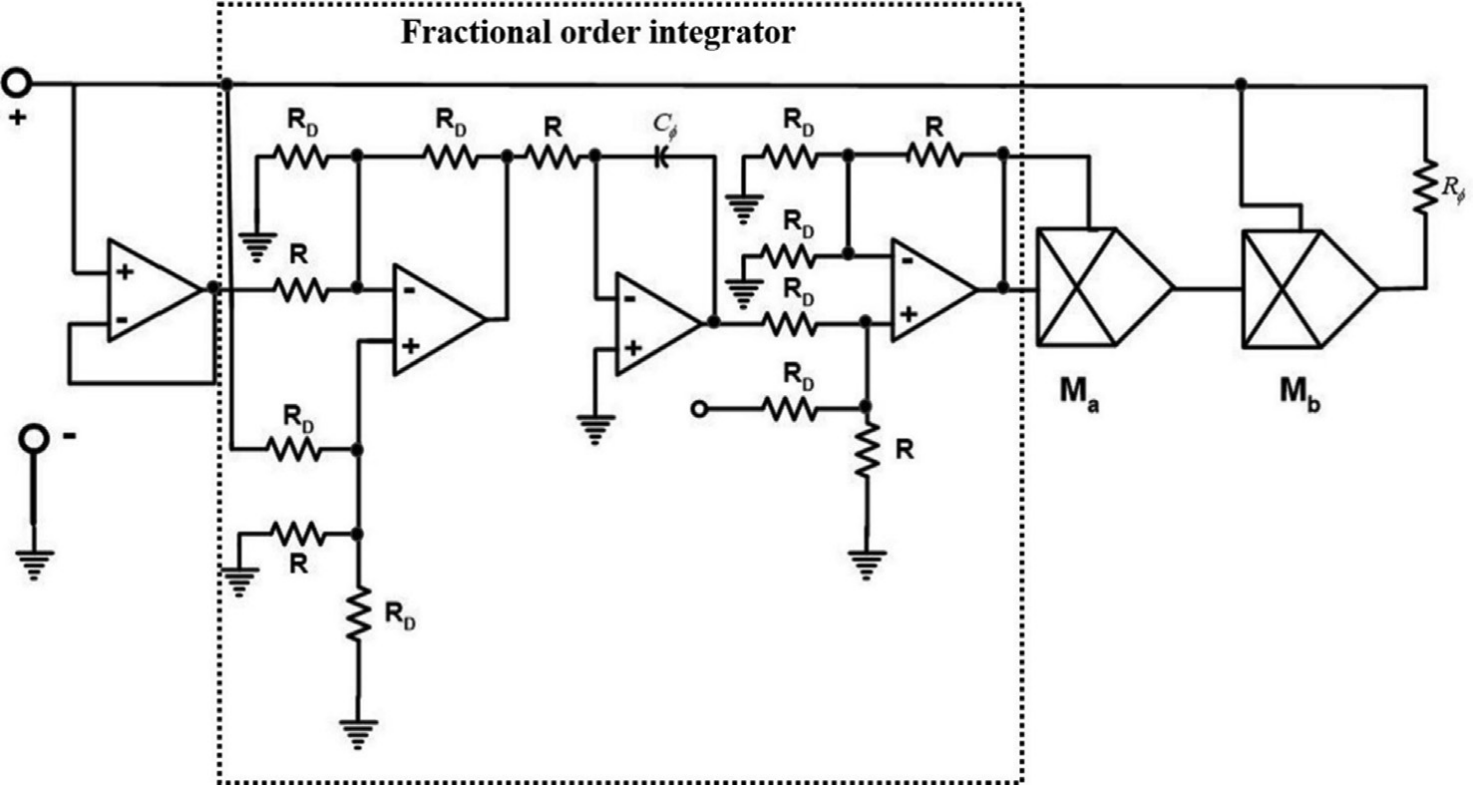
Memristor emulator with the fractional-order integrator.

**Fig. 2:**
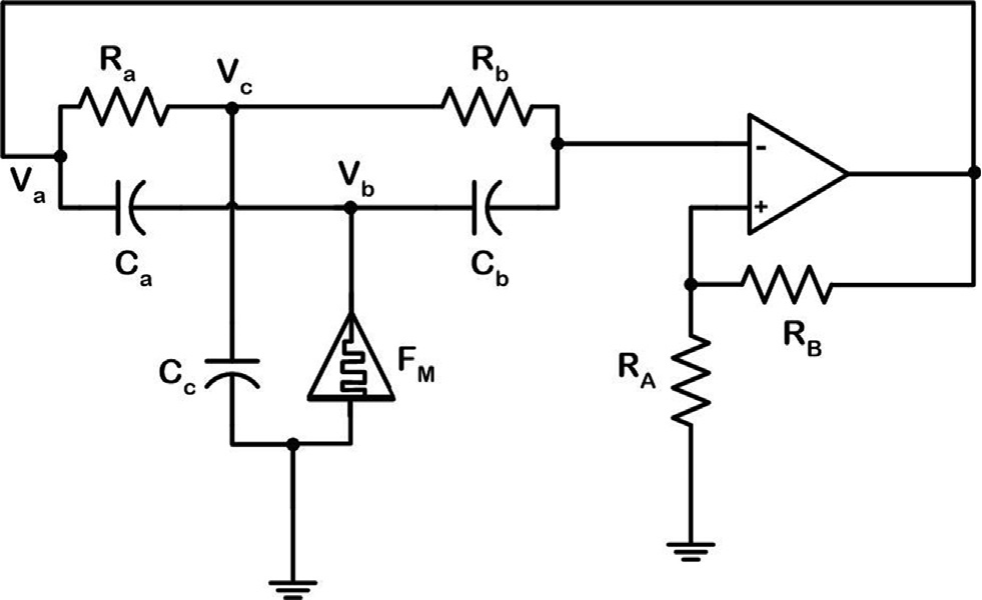
Twin-T oscillator with fracmemristor ($F_{M}$).

The oscillator, which is considered in this paper, is Twin-T memristor oscillator [29]. Unlike most of the fractional-order systems which consider all the elements as fractional ones, we just study the effect of the fractional-order memristor in integer-order Twin-T oscillator. In [29], the authors proposed a memristor emulator which contains an op-amp based integer-order integrator. We replace the integer-order integrator with the fractional-order one discussed in [30]. Fig. 1 shows the fracmemristor emulator, and Fig. 2 shows the Twin-T oscillator with this fracmemristor.

In Fig. 1, the value of the resistors is *R_D_* = *A*^–1^*R* where $A^{-1}=\frac{1+q}{1-q}$ and *q* represents the fractional order of the system [30]. The voltage-current relationship of the memristor emulator with fractional-order integrator will be

(2) $$\begin{array}{c} i=M\left(V_{\phi }\right)V=\frac{V-gV\left(gV_{\phi }^{2}\right)}{R_{\phi }}=\frac{1}{R_{\phi }}\left(1-g^{2}V_{\phi }^{2}\right)V\\ \frac{d^{\alpha }V_{\phi }}{dt^{\alpha }}=-\frac{V_{\phi }}{R_{D}C_{\phi }}-\frac{V}{RC_{\phi }} \end{array}$$

where *M*(*V_ϕ_*) is a continuous linear impedance function related to the voltage of the memristor *V_ϕ_* and equals $M\left(V_{\phi }\right)=\frac{1}{R_{\phi }}\left(1-g^{2}V_{\phi }^{2}\right)$.

Using KVL in Fig. 2, we can derive the dimensionless model [29] as

(3) $$\begin{array}{c} \dot{x}=a_{1}M\left(w\right)y+a_{2}z+a_{3}x,\;\dot{y}=a_{4}M\left(w\right)y+a_{5}z+a_{6}x,\;\dot{z}=a_{7}x+a_{8}z,\\ D^{q}w=a_{9}y+a_{10}w \end{array}$$

where $M\left(w\right)=\alpha +\beta w^{2}$, *x* = *V_a_*, *y* = *V_b_*, *z* = *V_c_* and *w* = *V_ϕ_*.

In this article, we used the Predict Evaluate Correct Evaluate (PECE) method of ABM, which its convergence and accuracy are discussed in [31]. To use the PECE method, we first consider a fractional-order dynamical system as

(4) $$D^{q}x=f\left(t,x\right),0\leq t\leq T$$

where $x^{k}\left(0\right)=x_{0}^{k}$for *k* ∈ [0, *n*–1]. This equation is analogous to the Volterra integral equation as

(5) $$x\left(t\right)=\sum_{k=0}^{n-1}x_{0}^{k}\frac{t^{k}}{k!}+\frac{1}{\Gamma \left(q\right)}\underset{0}{\int^{t}}\frac{f\left(\tau ,x\right)}{{\left(t-\tau \right)}^{1-q}}d\tau$$

which can be discretized as

(6) $$x_{h}\left(t_{n+1}\right)=\sum_{k=0}^{n-1}x_{0}^{\left(k\right)}\frac{t_{n}^{k+1}}{k!}+\frac{h^{q}}{\Gamma \left(q+2\right)}f\left(t_{n+1},x_{h}^{p}\left(t_{n+1}\right)\right)+\frac{h^{q}}{\Gamma \left(q+2\right)}\sum a_{j,n+1}f\left(t_{j},x_{h}\left(t_{j}\right)\right)$$

wherein (6), $h=\frac{T}{N}$and $t_{n}=nh$ as *h* ∈ [0, *N*]. Also, we have

(7) $$\begin{array}{c} a_{j,n+1}=\left\{\begin{array}{cc} n^{q+1}-\left(n-q\right){\left(n+1\right)}^{q+1}, & j=0\\ -2{\left(n-j+1\right)}^{q+1}, & 1\leq j\leq n\\ 1, & j=n+1 \end{array}\right)\\ x_{h}^{p}\left(t_{n+1}\right)=\sum_{k=0}^{n-1}x_{0}^{\left(k\right)}\frac{t_{n}^{k+1}}{k!}+\frac{h^{q}}{\Gamma \left(2\right)}\sum_{j=0}^{n}b_{j,n+1}f\left(t_{j}x_{h}\left(t_{j}\right)\right)\\ b_{j,n+1}=\frac{h^{q}}{q}\left({\left(n-j+1\right)}^{q}-{\left(n-j\right)}^{q}\right) \end{array}$$

The estimated error is $e=Max\left|x\left(t_{i}\right)-x_{h}\left(t_{i}\right)\right|=0\left(h^{p}\right)$ while j=0,1,⋯,N and p=Min(2,1+q).

Using the above, the fourth state of the FTT discrete form is

(8) $$w_{n+1}=\left\{\begin{array}{c} w_{0}+\frac{h^{q}}{\Gamma \left(q+2\right)}\left[a_{9}y_{n+1}^{p}+a_{10}w_{n+1}^{p}\right]\\ +\frac{h^{q}}{\Gamma \left(q+2\right)}\sum_{j=0}^{n}\left[\eta_{j,n+1}\left[a_{9}y_{j}+a_{10}w_{j}\right]\right] \end{array}\right\}$$

as

(9) $$w_{n+1}^{p}=w_{0}+\frac{1}{\Gamma \left(q+2\right)}\sum_{j=0}^{n}\omega_{j,n+1}\left[a_{9}y_{j}+a_{10}w_{j}\right]$$

and

(10) $$\begin{array}{c} \eta_{l,j,n+1}=\left\{\begin{array}{cc} n^{q+1}-\left(n-q\right){\left(n+1\right)}^{q+1}, & j=0\\ {\left(n-j+2\right)}^{q+1}+{\left(n-j\right)}^{q+1}-2{\left(n-j+1\right)}^{q+1}, & 1\leq j\leq n\\ 1, & j=n+1 \end{array}\right)\\ \omega_{l,j,n+1}=\frac{h^{q}}{q}\left[\left({\left(n-j+1\right)}^{q}-{\left(n-j\right)}^{q}\right),0\leq j\leq n\right] \end{array}$$

where *l* = 1.

To solve the equation, the fourth-order Runge-Kutta method is used for the first three states, and PECE is used for the fractional-order state in (3). Eq. (3) can be discretized as

(11) $$\begin{array}{c} x\left(n+1\right)=x\left(n\right)+\frac{1}{6}\left[K_{x}^{\left(1\right)}\left(n\right)+2K_{x}^{\left(2\right)}\left(n\right)+2K_{x}^{\left(3\right)}\left(n\right)+K_{x}^{\left(4\right)}\left(n\right)\right]\\ y\left(n+1\right)=y\left(n\right)+\frac{1}{6}\left[K_{y}^{\left(1\right)}\left(n\right)+2K_{y}^{\left(2\right)}\left(n\right)+2K_{y}^{\left(3\right)}\left(n\right)+K_{y}^{\left(4\right)}\left(n\right)\right]\\ \begin{array}{c} z\left(n+1\right)=z\left(n\right)+\frac{1}{6}\left[K_{z}^{\left(1\right)}\left(n\right)+2K_{z}^{\left(2\right)}\left(n\right)+2K_{z}^{\left(3\right)}\left(n\right)+K_{z}^{\left(4\right)}\left(n\right)\right]\\ w\left(n+1\right)=\left\{\begin{array}{c} w\left(n\right)+\frac{h^{q}}{\Gamma \left(q+2\right)}\left[a_{9}y_{n+1}^{p}+a_{10}w_{n+1}^{p}\right]\\ +\frac{h^{q}}{\Gamma \left(q+2\right)}\sum_{j=0}^{n}\left[\eta_{j,n+1}\left[a_{9}y_{j}+a_{10}w_{j}\right]\right] \end{array}\right\} \end{array} \end{array}$$

where

(12) $$\begin{array}{c} K_{x}^{\left(1\right)}\left(n\right)=hf_{x}\left[x\left(n\right),y\left(n\right),z\left(n\right),w\left(n\right)\right]\\ K_{x}^{\left(2\right)}\left(n\right)=hf_{x}\left[x\left(n\right)+\frac{K_{x}^{\left(1\right)}\left(n\right)}{2},y\left(n\right)+\frac{K_{y}^{\left(1\right)}\left(n\right)}{2},z\left(n\right)+\frac{K_{z}^{\left(1\right)}\left(n\right)}{2}+\frac{K_{w}^{\left(1\right)}\left(n\right)}{2}\right]\\ \begin{array}{c} K_{x}^{\left(3\right)}\left(n\right)=hf_{x}\left[x\left(n\right)+\frac{K_{x}^{\left(2\right)}\left(n\right)}{2},y\left(n\right)+\frac{K_{y}^{\left(2\right)}\left(n\right)}{2},z\left(n\right)+\frac{K_{z}^{\left(2\right)}\left(n\right)}{2}+\frac{K_{w}^{\left(2\right)}\left(n\right)}{2}\right]\\ K_{x}^{\left(4\right)}\left(n\right)=hf_{x}\left[x\left(n\right)+\frac{K_{x}^{\left(3\right)}\left(n\right)}{2},y\left(n\right)+\frac{K_{y}^{\left(3\right)}\left(n\right)}{2},z\left(n\right)+\frac{K_{z}^{\left(3\right)}\left(n\right)}{2}+\frac{K_{w}^{\left(3\right)}\left(n\right)}{2}\right] \end{array} \end{array}$$

**Fig. 3:**
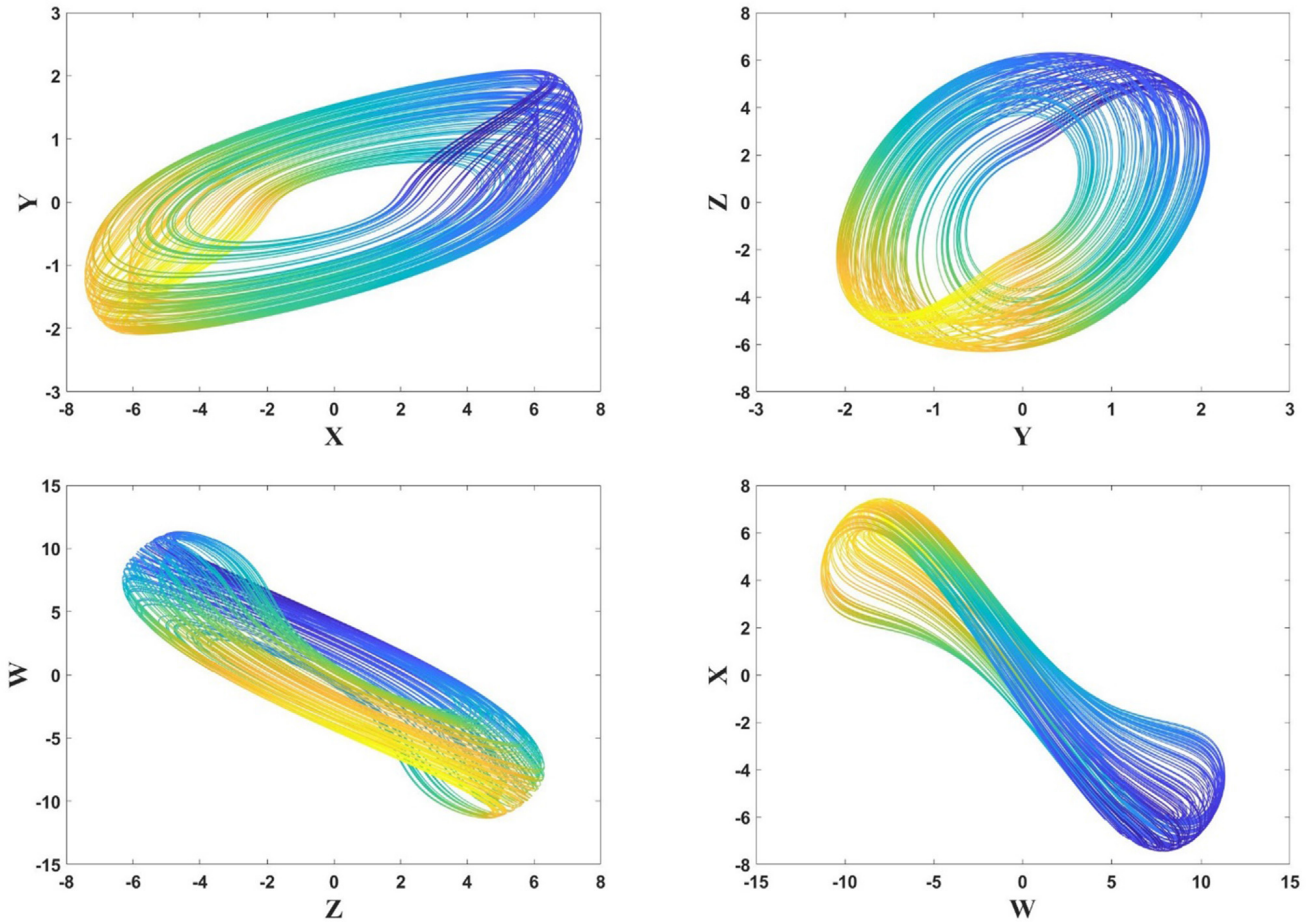
The phase portraits of the FTT system in (x-y), (y-z), (z-w) and (w-x) plane when $a_{1}=9$, $a_{2}=-0.77$, $a_{3}=0.07$, $a_{4}=0.75$, $a_{5}=-0.42$, $a_{6}=0.0382$, $a_{7}=3.532$, $a_{8}=-3.85$, $a_{9}=-10$, $a_{10}=-1$, α=1 , β=-0.01, and q=0.99.

Similarly, the Runge-Kutta coefficients for the other two states (*y*, *z*) can be calculated as (12). For the parameter values of $a_{1}=9$, $a_{2}=-0.77$, $a_{3}=0.07$, $a_{4}=0.75$, $a_{5}=-0.42$, $a_{6}=0.0382$, $a_{7}=3.532$, $a_{8}=-3.85$, $a_{9}=-10$, $a_{10}=-1$, α=1 , β=-0.01 and q=0.99, the 2D phase portraits of the FTT system are shown in Fig. 3.

## Analysis of the FTT oscillator

Equilibrium points, corresponding eigenvalues, stability, LEs, and bifurcation diagram of the FTT are examined to the system in this section.

### Statistical analysis of the system

The FTT system shows three fixed points as below

(13) $$E_{1}=[0,0,0,0],\;E_{2}=\left[0,,,-,\frac{a_{10}}{a_{9}},\sqrt{\frac{-\alpha }{\beta }},,,0,,,\sqrt{\frac{-\alpha }{\beta }}\right],\;E_{3}=\left[0,,,\frac{a_{10}}{a_{9}},\sqrt{\frac{-\alpha }{\beta }},,,0,,,-,\sqrt{\frac{-\alpha }{\beta }}\right]$$

The Jacobian matrix of the FTT system is

(14) $$J\left(X\right)=\left|\begin{array}{cccc} a_{3} & a_{1}\left(\beta w^{2}+\alpha \right) & a_{2} & 2a_{1}\beta wy\\ a_{6} & a_{4}\left(\beta w^{2}+\alpha \right) & a_{5} & 2a_{4}\beta wy\\ a_{7} & 0 & a_{8} & 0\\ 0 & a_{9} & 0 & a_{10} \end{array}\right|$$

The equation $\det(diag\left(\lambda^{M_{q1}},\lambda^{M_{q2}},\lambda^{M_{q3}},\lambda^{M_{q4}}\right)-J_{E_{i}})=0$ yields the generalized characteristic polynomial of the FTT system. In this equation, $q_{1}=q_{2}=q_{3}=1$, $q_{4}=0.99$ and M is the least common multiple (LCM) of $q_{i}$for i=1,⋯,4. The characteristic equations at $E_{1},E_{2}$ and $E_{3}$ are given by (15), (16), (17) respectively.

(15) $$\begin{array}{c} \lambda^{399}+\lambda^{300}+3.03\lambda^{299}+3.03\lambda^{200}-0.72866\lambda^{199}-0.72866\lambda^{100}\\ +10.189725\lambda^{99}+10.189725=0 \end{array}$$

(16) $$\lambda^{399}+\lambda^{300}+3.78\lambda^{299}+5.28\lambda^{200}+2.45014\lambda^{199}+8.80774\lambda^{100}-20.37945=0$$

(17) $$\lambda^{399}+\lambda^{300}+3.78\lambda^{299}+5.28\lambda^{200}+2.45014\lambda^{199}+8.80774\lambda^{100}-20.37945=0$$

Corollary 1. *The fixed points should be unstable to the FTT system exhibit chaotic dynamics. So the essential condition is any λ of the equilibrium points should satisfy the following inequality*

(18) $$q>\frac{2}{\pi }arctan\left(\frac{\left|Im\left(\lambda \right)\right|}{Re\left(\lambda \right)}\right)$$

The eigenvalues of the FTT at the equilibrium $E_{1}$ when a=3are *λ*_1,2_ = 0.5000 ± 0.8660i and *λ*_3_ = –2, which to satisfy (18), we have *q* > 0.97.

Corollary 2. *A chaotic attractor exists in the FTT if the corresponding equilibrium points show instability. So the essential condition is that the roots of the characteristic equations* (15)*,* (16), (17) *should satisfy the following inequality*

(19) $$\frac{\pi }{2M}-\min_{i}\left\{arg\left(\lambda_{i}\right)\right\}\geq 0$$

It can be concluded from [32] that the system is unstable as not all the roots of the equations (15), (16), (17) satisfy the condition (19). Hence, we can conclude the existence of chaotic oscillations like its integer-order system discussed in [29] when *q* > 0.97.

### Lyapunov exponents

**Fig. 4:**
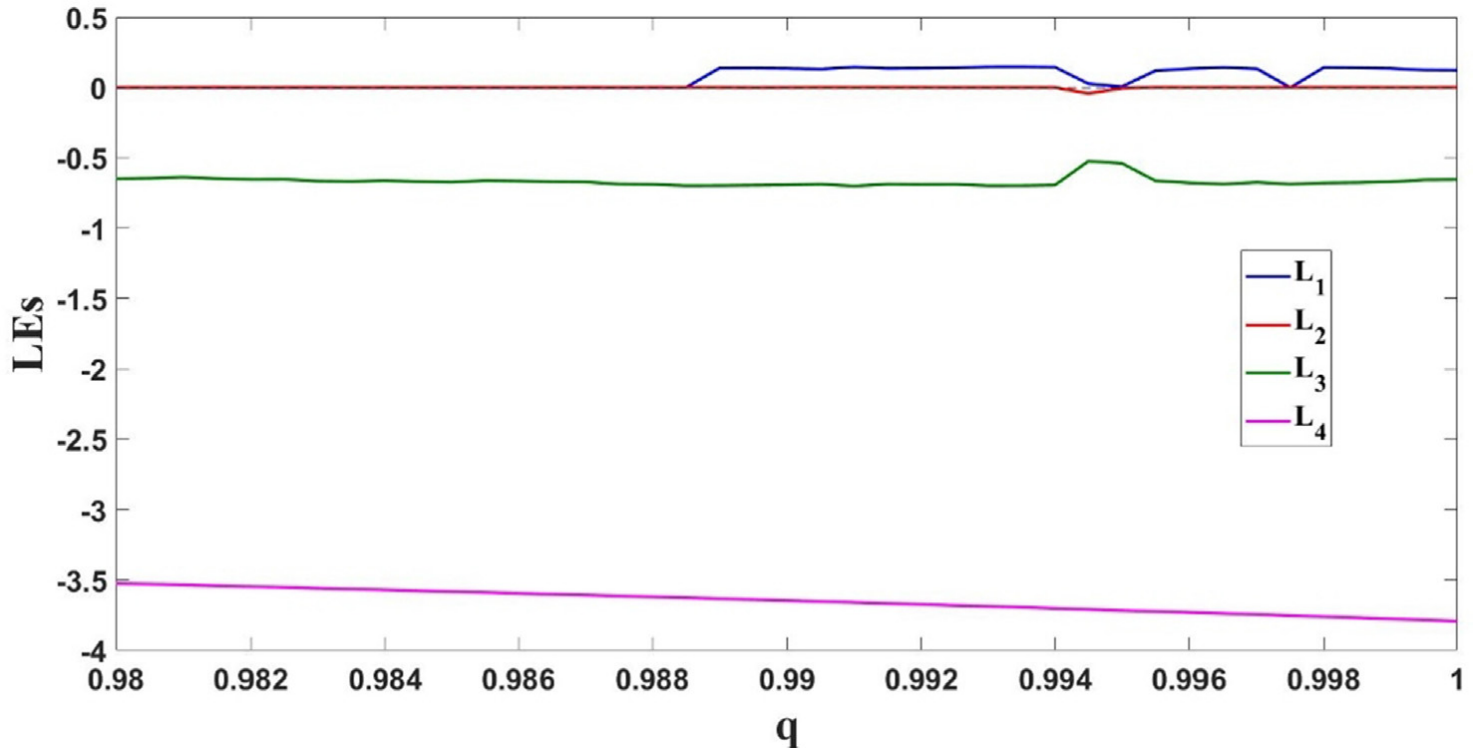
Lyapunov exponents of the FTT system as q increases. This fig. shows that the system exhibits different responses.

Wolfs algorithm is used to derive the Lyapunov exponents of the FTT system and check the chaoticity of the system for different values of the parameters. Also, the fractional-order predictor–corrector solver fde12 is used instead of the ordinary differential equation (ODE) solvers [33]. The Lyapunov exponents of the FTT system for different values of the fractional order q are shown in Fig. 4.

### Bifurcation diagram

**Fig. 5:**
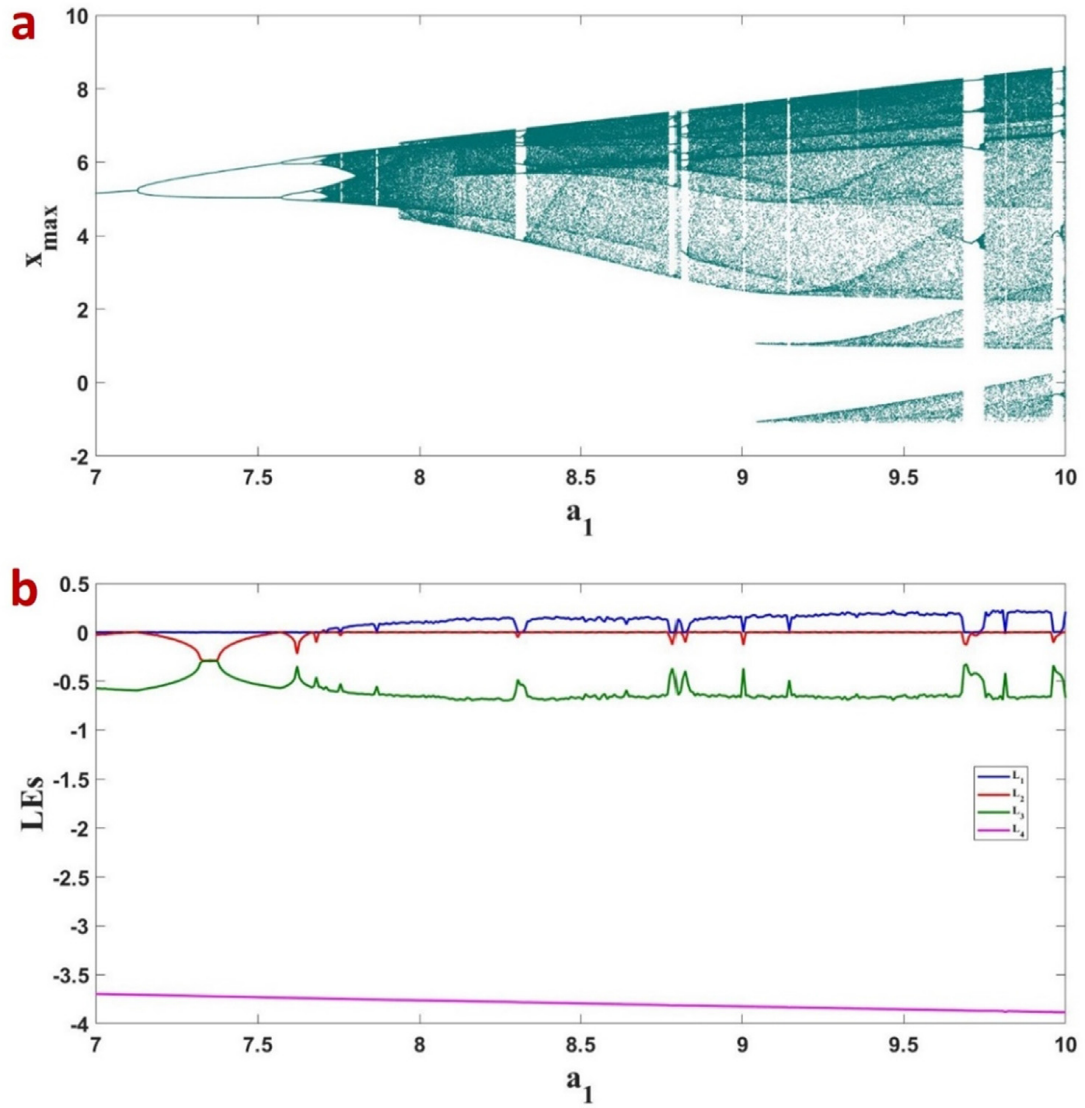
a) The bifurcation plot of the FTT versus the parameter$a_{1}$ and b) the corresponding LEs.

**Fig. 6:**
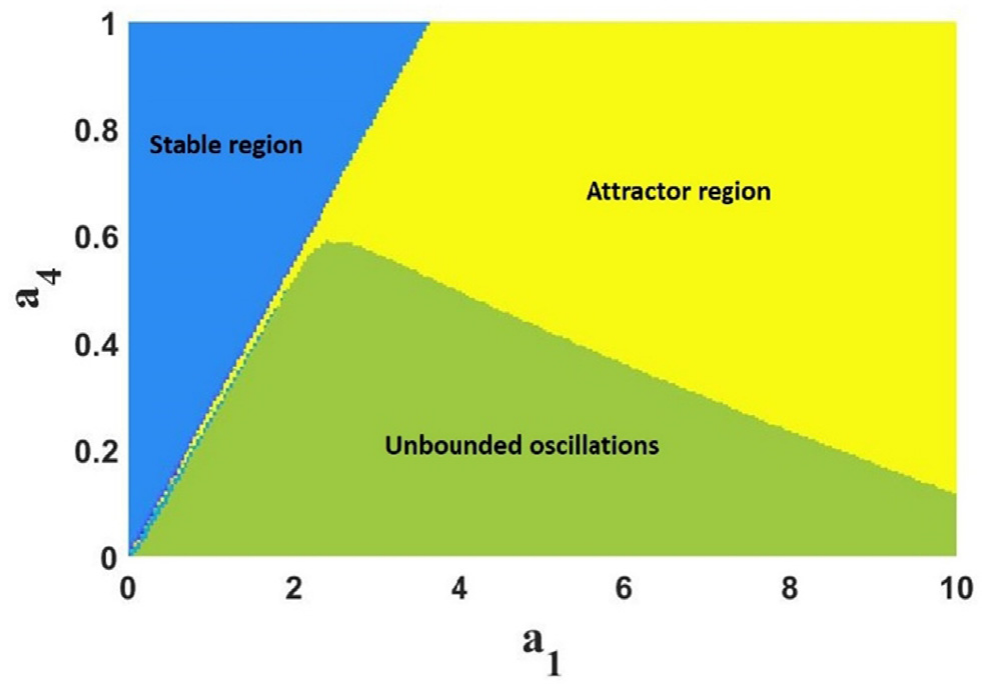
2D bifurcation diagram for $a_{1}$and $a_{4}$ when the fractional-order equals 0.99.

To investigate the impact of the parameters on the FTT oscillator, we derived the bifurcation plots where we plotted the local maxima of the state variables versus the control parameter. We have considered $a_{1}$ as the bifurcation parameter and the local maxima of x in Fig. 5a. The FTT takes a period-doubling route to the chaos, which is similarly supported by the Lyapunov exponents shown in Fig. 5b. The fractional order for the bifurcation plot is taken as q=0.99, and the other parameters are considered as used in Fig. 3. Also, to show the effect of the parameters $a_{4}$ and $a_{1}$, the 2D bifurcation diagram of the system is plotted in Fig. 6. This figure shows the different ranges of the parameters which yield stable equilibrium, strange attractor, and unbounded responses.

### Multistability

**Fig. 7:**
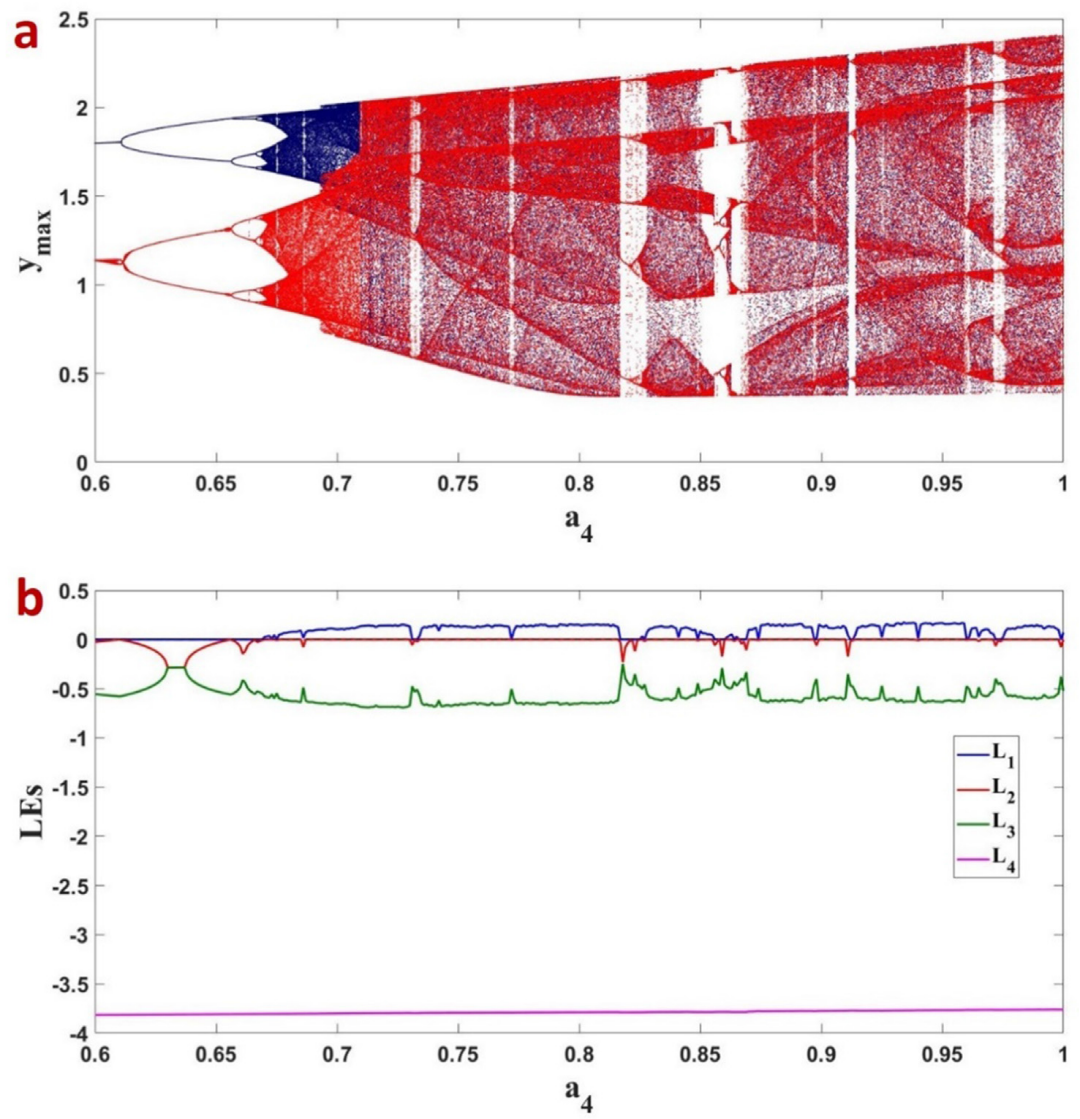
a) The bifurcation plot of the FTT versus $a_{4}$ which forward and backward are shown in blue and red dots, respectively. b) The corresponding LEs are also plotted.

**Fig. 8:**
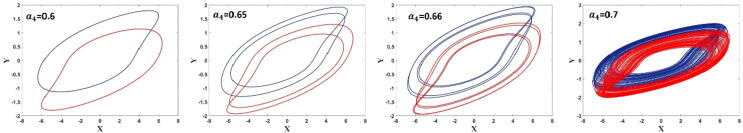
Various coexisting limit cycles and chaotic attractors when the initial conditions are $\left[1,0,0,0\right]$ (shown in blue) and $\left[-1,0,0,0\right]$ (shown in red) for different values of $a_{4}$.

To study the multistability, the forward (parameter increases) and backward (parameter decreases) bifurcations are considered. The initial condition for each parameter is the final value of the trajectory in the previous parameter. In Fig. 7, parameter $a_{4}$ is the bifurcation parameter, and the local maxima of the state variable yare plotted when the fractional order equals q=0.99.Fig. 7a shows the bifurcation of the FTT system while the forward and backward shown in blue and red, respectively. Fig. 7b shows the corresponding LEs. We could see the coexistence of chaotic attractors for $0.6694\leq a_{4}\leq 0.7092$, period-8 limit cycles for $0.6568\leq a_{4}\leq 0.6664$ and period-4 limit cycles for $0.6105\leq a_{4}\leq 0.6567.$ The various coexisting attractors for different values of the parameter $a_{4}$ are shown in Fig. 8.

**Fig. 9:**
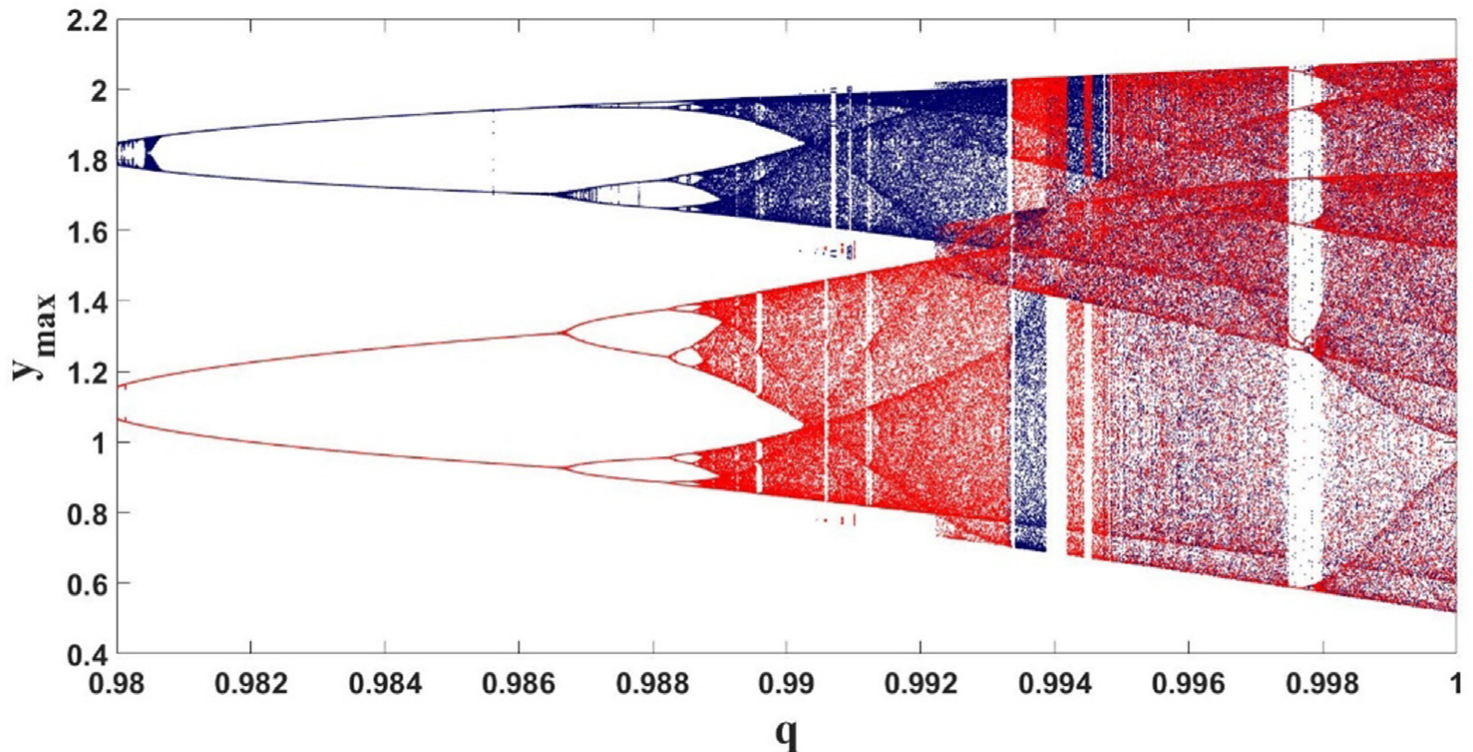
The bifurcation plot of the FTT versus q when forward and backward continuations are shown in blue and red, respectively, which shows coexisting attractors in this system.

**Fig. 10:**
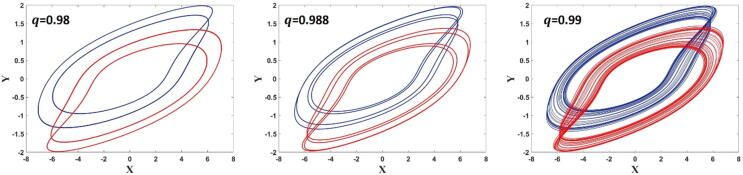
Various coexisting limit cycles and strange attractors when the initial conditions are set to $\left[1,0,0,0\right]$ (shown in blue) and $\left[-1,0,0,0\right]$ (shown in red) for different values of q.

We use the same forward and backward continuation to check the multistability and coexisting attractors for the fractional order q. Also, the other parameters are considered as used for Fig. 3. We could identify the coexistence of period-2 limit cycles for 0.98≤q≤0.9867, period −4 limit cycles for 0.9868≤q≤0.9883, and chaotic attractors for 0.9887≤q≤0.9948 as seen in Fig. 9. Fig. 10 shows the various coexisting limit cycles and chaotic attractors for different values of the fractional orderq.

**Fig. 11:**
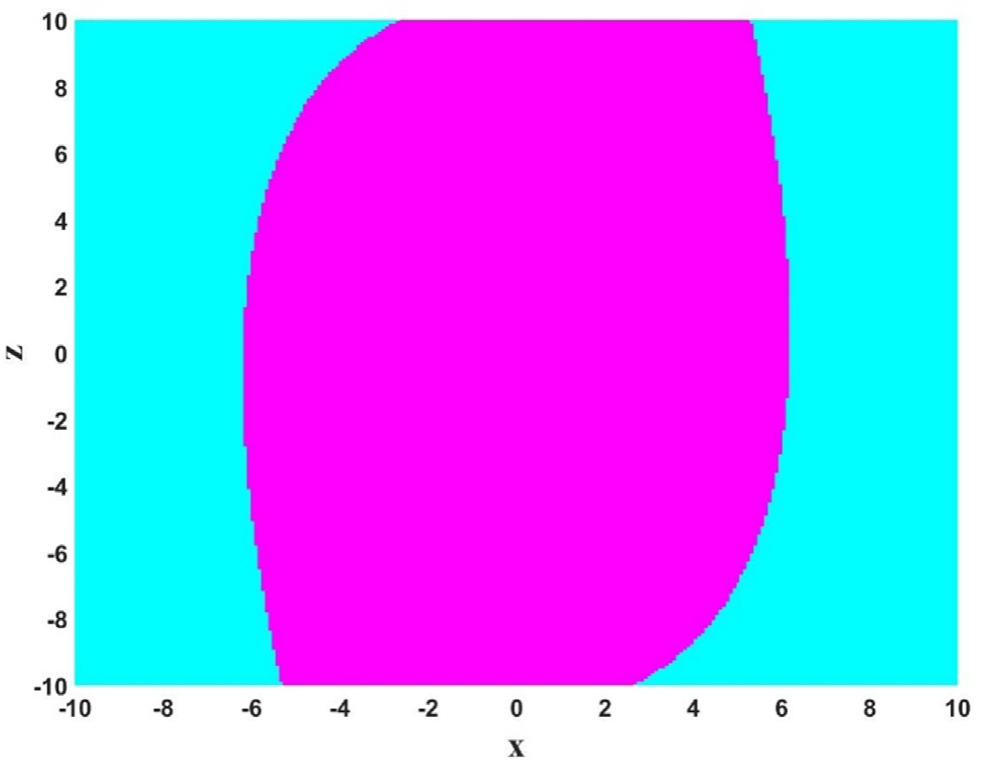
Basin of attraction of the system in the x-z plane when y(0) = 0 and w(0) = 0. In this figure, cyan and magenta color show unbounded and chaotic responses.

To better analyze the coexisting attractors of the system, the Basin of attraction of the system is considered in the x-z plane when y(0) = 0 and w(0) = 0. In Fig. 11, cyan and magenta color show unbounded and chaotic responses of the system, respectively.

### Antimonotonicity

**Fig. 12:**
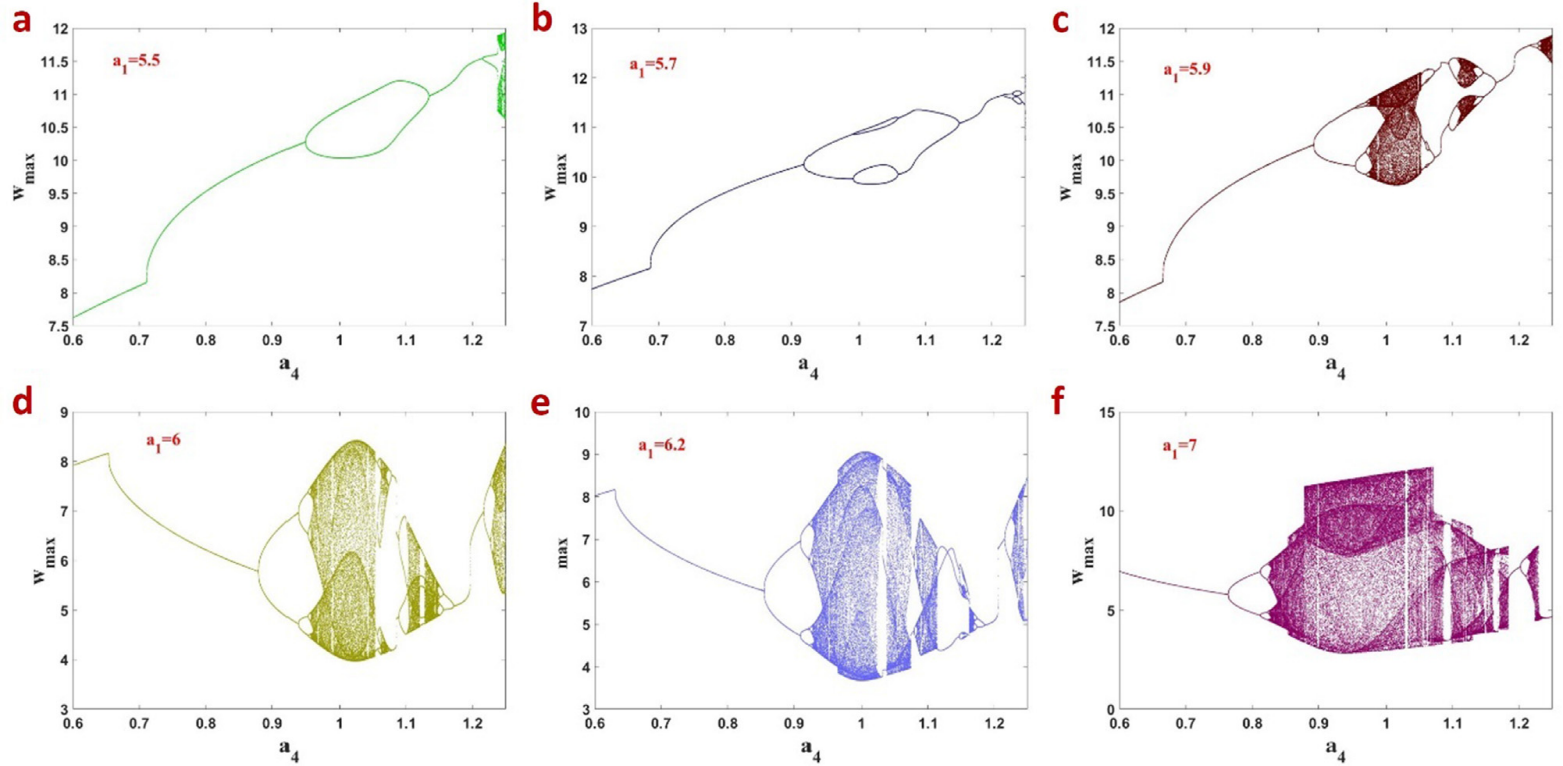
Bifurcation of the FTT oscillator with $a_{4}$ for q=0.99 and different fixed values of $a_{1}$ which claims existence of antimonotonicity in this system.

Antimonotonicity, a complex behavior in nonlinear systems, means the occurrence of period-doubling and inverse period-doubling. In the bifurcation diagram of these systems, the periodic attractors double as parameter increases and instantly joining periodic attractors form smaller ones, so emerging antimonotonicity. To examine antimonotonicity, the bifurcation of the FTT oscillator system is considered as $a_{4}$ increases while the fractional-order q=0.99 and parameter $a_{1}$ has some different fixed values (Fig. 12).

## Conclusion

To investigate memory-dependent systems and consider history in the electronic circuit, we can use the memristor element. In this article, we showed that using fractional-order memristor in an integer-order oscillator circuit enables the system to show complex behaviors. For example, we concluded and showed that in some range of the fractional order, q>0.97, the system can show chaotic responses. Multistability, the existence of two or more attractors for a fixed value of the parameter, and antimonotonicity, the existence of period-doubling route to chaos and inverse of it, are the properties that this system shows in different value of the parameters. Precise ranges of the parameters are derived using the bifurcation diagram or its corresponding Lyapunov exponents. We also use a 2D bifurcation diagram to show the different attractors of the system as two different controlling parameters change.

## Compliance with ethics requirements

This article does not contain any studies with human or animal subjects

## Declaration of Competing Interest

The authors have declared no conflict of interest
